# Neoadjuvant chemotherapy for pineal region tumors

**DOI:** 10.1007/s11060-026-05638-9

**Published:** 2026-06-03

**Authors:** Christopher Troy, David G. Laird, Cameron Brimley, Soniya Pinto, Sean Himel, Carlos Osorno-Cruz, Mustafa Motiwala, Emal Lesha, Kelly Chamberlin, Giles Robinson, Amar Gajjar, Jason Chiang, Nir Shimony, David S. Hersh, Paul Klimo

**Affiliations:** 1 Department of Neurosurgery, University of Tennessee Health Science Center, Semmes Murphey Clinic, 6325 Humphreys Blvd., Memphis, TN 38120 USA; 2 College of Medicine, University of Tennessee Health Science Center (UTHSC), Memphis, TN USA; 3 Geisinger Janet Weis Children’s Hospital, Danville, PA USA; 4 Department of Radiology, St. Jude Children’s Research Hospital, Memphis, TN USA; 5 Le Bonheur Children’s Hospital, Memphis, TN USA; 6 Department of Oncology, St. Jude Children’s Research Hospital, Memphis, TN USA; 7 Department of Pathology, St. Jude Children’s Research Hospital, Memphis, TN USA; 8 Neuroscience Institute, Le Bonheur Children’s Hospital, Memphis, TN USA; 9 Division of Surgery, St. Jude Children’s Research Hospital, Memphis, TN USA; 10 Semmes Murphey, Memphis, TN USA; 11 Division of Neurosurgery, Connecticut Children’s, Hartford, CT USA; 12 Department of Neurosurgery, UConn School of Medicine, Farmington, CT USA; 13 Center for Child Health Equity Research and Innovative Outcomes, Connecticut Children’s Research Institute, Hartford, CT USA

**Keywords:** Neoadjuvant chemotherapy, Pineal region tumors, Pediatrics

## Abstract

**Objective:**

Pineal region tumors are frequently large at diagnosis, often presenting with obstructive hydrocephalus. Surgical resection can be challenging due to intrinsic tumor vascularity and the surrounding eloquent neurovascular anatomy. For patients with large pineal region tumors, particularly young children, neoadjuvant chemotherapy may decrease both the size and vascularity of the tumor before definitive resection. Herein we present our experience.

**Methods:**

A single-center retrospective review of pediatric patients with pineal region/posterior third ventricular tumors was performed. Each patient received at least 2 rounds of neoadjuvant chemotherapy (carboplatin, etoposide, cyclophosphamide) prior to definitive resection. The primary outcome measure for this study was the radiographic effect of said chemotherapy on the tumor. Pre- and post-chemotherapy tumor volumes were calculated using magnetic resonance imaging tumor volume analytics. Secondary outcomes were estimated intraoperative blood loss, extent of resection, operative complications, readmission rates, and patient survival. Blood loss was calculated as a percentage of estimated total blood volume.

**Results:**

We identified thirty-one children and young adults (16 males, 15 females) who underwent neoadjuvant chemotherapy for pineal region tumors. The mean age of the population was 7.42 years (5 mo–22 yr). Pathologies were predominantly embryonal and included pineoblastoma (n=22; 71%), atypical teratoid rhabdoid tumor (n=5; 16%), pineal parenchymal tumor of intermediate differentiation (n=3; 10%), and embryonal tumor with multilayered rosettes (n=1; 3%). The average percent tumor volume reduction post chemotherapy was 51% (range, -12.4–90.0%); only 1 patient had an increase in tumor volume. There were no complications related to chemotherapy. All surgeries resulted in gross total or near total resections. Average blood loss as a percentage of preoperative total blood volume was 11.7% (1.1–38.8%). Average length of follow-up was 6.33 years (range, 1–14 years) with 20 patients (64.5%) alive at last follow-up.

**Conclusions:**

Neoadjuvant chemotherapy for certain pineal region tumors is a safe and effective means to decrease tumor volume. While we believe this reduction in tumor size facilitates subsequent resection, further multicenter studies are needed.

## Introduction

Tumors of the pineal region are relatively rare, representing 3–11% of all pediatric brain tumors [1–3]. Germ cell and pineal parenchymal tumors are the main lineages found in children [2]. Patients commonly present with obstructive hydrocephalus but may also have symptoms secondary to mass effect on surrounding structures, such as the dorsal midbrain or cerebellum. These tumors, particularly in young children, may be quite large and vascularized at the time of diagnosis [2, 4].

Pineal tumors in children require a multimodal approach integrating clinical, radiographic, and laboratory data [5]. High resolution magnetic resonance imaging (MRI) defines the tumor size, extent, and relationship to surrounding venous anatomy, while cerebrospinal fluid (CSF) and serum tumor markers (alpha-fetoprotein [AFP], beta human chorionic gonadotropin [β-hCG]) may guide the diagnosis and obviate the need for a surgical biopsy in select cases [2]. If hydrocephalus is present, intraventricular endoscopy may provide a minimally invasive means to treat the hydrocephalus via endoscopic third ventriculostomy (ETV), which provides an opportunity to concurrently obtain CSF +/- tumor tissue for analysis [6, 7]. However, there are known and suspected risk factors for ETV failure such as young age, presence of metastatic disease, and malignant pathology [8–10]. Patel et al. reported a single center ETV failure rate of 42.3% in pineal tumor patients [11]. Alternatively, if hydrocephalus is not present, a lumbar puncture and craniotomy may be needed to obtain CSF and tumor tissue, respectively.

Management of pineal region tumors remains complex because of their histologic heterogeneity and variable response to chemotherapy and radiotherapy, notably in young children where treatment-related toxicities pose significant challenges [12, 13]. Gross or near total resection has been correlated with improved survival in certain tumor types without metastatic disease, as well as in a subset of children with metastatic disease [4, 14]. However, the deep-seated location and surrounding neurovascular structures of the pineal region make it demanding to access surgically. This can be compounded by large tumor size and hypervascularity [15]. To address these challenges, multidisciplinary, patient-tailored treatment strategies have been emphasized [13, 14].

Pre-resection (i.e., neoadjuvant) chemotherapy can reduce tumor vascularity, induce necrosis and cystic changes, and reduce overall tumor volume, thereby potentially improving the safety and feasibility of subsequent surgical resection. This strategy has been used effectively in choroid plexus carcinoma (CPC) and embryonal tumors [4, 16]. However, its utility for pineal region tumors has not been well characterized. Herein, we present our experience with neoadjuvant chemotherapy prior to definitive resection of pineal region tumors in a predominantly pediatric population.

## Methods

### Study design and population

Institutional review board approval was granted by the University of Tennessee Health Science Center, Memphis; patient consent was waived because of the retrospective design of the study. Patients were identified from the brain tumor database at Le Bonheur Children’s Hospital, Memphis, TN from 2015 to 2025. All patients who underwent resection of pineal region tumors were screened. We searched the electronic medical record system to determine age, tumor pathology, and whether patients underwent neoadjuvant therapy. Inclusion criteria were children and young adults with a confirmed tumor of the pineal region who received at least 2 rounds of neoadjuvant chemotherapy prior to attempted resection. Patients with prior partial resection or biopsy performed at an outside facility were included. As a group, germ cell tumors were excluded from this study as resection is not a standard part of their treatment (e.g., germinomatous and non-germinomatous germ cell tumors) or chemotherapy is ineffective (e.g., teratoma). We recognize that some pineal germ cell patients will undergo so-called 2nd look surgery to remove post-treatment residual abnormalities, but this is rare.

### Data collection

Demographic information, follow-up details, and pre-, intra-, and post-operative data were collected. The primary outcome of this study was the change in tumor volume after neoadjuvant chemotherapy. Additional outcomes were intraoperative blood loss, extent of resection (EOR), operative complications, readmission rates, and patient survival. Gross total resection (GTR) was defined as no demonstrable residual tumor on MRI; near total resection (NTR) was defined as > 90% tumor resection and subtotal resection (STR) was < 90%. Blood loss was calculated as a percentage of estimated total blood volume.

### Tumor volumetric analysis

Pre- and post-chemotherapy contrast-enhanced brain MRIs were uploaded onto a segmentation platform (Mint Medical, Hamilton NJ). Pineal tumors were segmented on axial 2D or 3D post-contrast T1sequences using a 3D segmentation tool by a board-certified radiologist with expertise in pediatric neuroimaging (SP). Tumor volumes, bi-perpendicular diameters and their percentage changes were automatically generated by the software.

### Treatment algorithm

Treatment begins with CSF diversion, if needed, and analysis of serum and CSF germ cell markers. When hydrocephalus is present, an ETV and concurrent endoscopic tumor biopsy is preferred, but a risk-benefit analysis needs to be undertaken first if the surgeon has concerns regarding the risk of tumor bleeding. If endoscopic biopsy was not performed or was non-diagnostic, then an open biopsy via craniotomy is strongly considered. Tissue diagnosis is always preferred prior to starting neoadjuvant chemotherapy, but in some circumstances, we will proceed without pathologic diagnosis (e.g., bleeding complication with prior non-diagnostic biopsy or imaging features that make the surgeon feel that biopsy-induced bleeding is a high risk).

All patients are discussed at a multidisciplinary tumor board. Neoadjuvant chemotherapy is considered in all patients, but particularly those with the following features: (1) imaging and pathology that show significant tumor vascularity; (2) large tumor size; and (3) young age that increases the risk of excessive blood volume loss. There are no specific age or tumor size parameters; these and other factors are collectively used to formulate a treatment recommendation. The goal of neoadjuvant chemotherapy is to reduce the size and vascularity of the tumor, and alter the consistency (e.g., necrosis) and composition (e.g., cystic transformation). Patients typically undergo 2 rounds of chemotherapy. Each round of chemotherapy typically proceeds as follows: day 1 includes carboplatin 560 mg/m^2^ IV and etoposide 100 mg/m^2^ IV; day 2 includes cyclophosphamide 1200 mg/m^2^ IV with mesna support and etoposide 100 mg/m^2^ IV [17, 18]. On day 3, granulocyte colony stimulating factor (5mcg/kg) is given for 10–14 days. Each cycle is 3–4 weeks in total duration. After 2 cycles of chemotherapy, the patient’s case is re-presented at our multidisciplinary conference where pre- and post-chemotherapy imaging is reviewed. A decision is made whether to continue with additional chemotherapy or proceed with definitive resection. Time from completing chemotherapy to surgery averages about 2 weeks, being primarily a function of recovery of blood counts and operating room availability.

## Results

### Demographic information and prior surgeries

A total of 31 patients with pineal region tumors underwent neoadjuvant chemotherapy followed by tumor resection (Table 1). There were 16 (51.6%) males and 15 (48.4%) females with a mean age on the date of surgery of 7.42 years (range, 5 months–22 years). Twenty-nine patients were under the age of 18, but there were 2 patients older than 18 years (19 and 22-year-old). Most patients were Caucasian (71.0%). Eleven (35.5%) patients had an existing shunt, 21 (67.7%) patients had a prior ETV, 25 (80.6%) patients had a prior biopsy, and 3 (9.7%) patients had a prior subtotal resection. Three patients did not have pathologic diagnosis prior to resection as it was felt there was a significant risk of bleeding with endoscopic biopsy; the imaging in these 3 patients were suggestive of an embryonal-type tumor.

**Table 1:** Population information (*N* = 31)

Characteristic	Value
Patients, n (%)	
Male	16 (51.6)
Female	15 (48.4)
Age, mean (range)	7.42 (5 mo–22 year)
Race, n (%)	
Caucasian	22 (71.0)
African American	7 (22.6)
Hispanic	1 (3.2)
Other/unspecified	1 (3.2)
Prior surgical history, n (%)	
Existing shunt	11 (35.5)
ETV	21 (67.7)
Biopsy	25 (80.6)
Subtotal resection	3 (9.7)
Tumor type, n (%)	
Pineoblastoma	22 (71.0)
ATRT	5 (16.1)
PPTID	3 (9.7)
ETMR	1 (3.2)
Chemotherapy rounds, n (%)	
2	27 (87.1)
3	4 (12.9)
Extent of resection, n (%)	
GTR	20 (64.5)
NTR	11 (35.5)

Abbreviations: ATRT=atypical teratoid/rhabdoid tumors; ETMR=embryonal tumors with multilayered rosettes; ETV=endoscopic third ventriculostomy; GTR=gross total resection; NTR=near total resection; PPTID=pineal parenchymal tumors of intermediate differentiation

### Tumor pathology and volumetric analysis

The most common type of tumor was pineoblastoma (*n* = 22; 71.0%), followed by atypical teratoid/rhabdoid tumors (ATRT) (*n* = 5; 16.1%), pineal parenchymal tumors of intermediate differentiation (PPTID) (*n* = 3; 9.7%), and embryonal tumors with multilayered rosettes (ETMR) (*n* = 1; 3.2%) (Table 2). Most patients received 2 rounds of neoadjuvant chemotherapy (*n* = 27, 87.1%); the remaining (*n* = 4, 12.9%) received 3 rounds. There were no documented complications related specifically to neoadjuvant chemotherapy, including infectious or hemorrhagic complications.

**Table 2:** Tumor volume analytics and blood loss information

Analytic	Overall (*N* = 31) mean (range)	PB (*n* = 22) mean (range)	ATRT (*n* = 5) mean (range)	PPTID (*n* = 3) mean (range)	ETMR (*n* = 1)
Pre-chemotherapy tumor volume, cm^3^	24.7 (1.1–84.1)	22.1 (3.5–81.5)	39.5 (1.1–84.1)	16.6 (5.0–37.3)	30.1
Post-chemotherapy tumor volume, cm^3^	12.1 (0.5–54.4)	11.1 (2.1–54.5)	17.9 (1.0–36.8)	9.8 (0.5–26.7)	13.0
Absolute volume reduction, cm^3^	12.6 (-6.0–65.5)	11.0 (-6.0–65.6)	21.6 (0.1–47.4)	6.8 (4.5–10.6)	17.1
Percent volume reduction	51.0 (-12.3–90.0)	49.8 (-12.4–84.2)	54.7 (10.53–74.5)	41.2 (28.4–90.0)	56.9
Estimated blood lost, mL	180 (25–900)	182 (25–900)	175 (100–250)	125 (25–200)	275
Percent total blood volume loss	11.7 (1.1–38.8)	9.3 (1.1–38.8)	21.6 (14.81–33.0)	6.4 (1.7–10.1)	31.3

Abbreviations: ATRT=atypical teratoid/rhabdoid tumors; ETMR=embryonal tumors with multilayered rosettes; GTR=gross total resection; NTR=near total resection; PB=Pineoblastoma; PPTID=pineal parenchymal tumors of intermediate differentiation

**Fig. 1:**
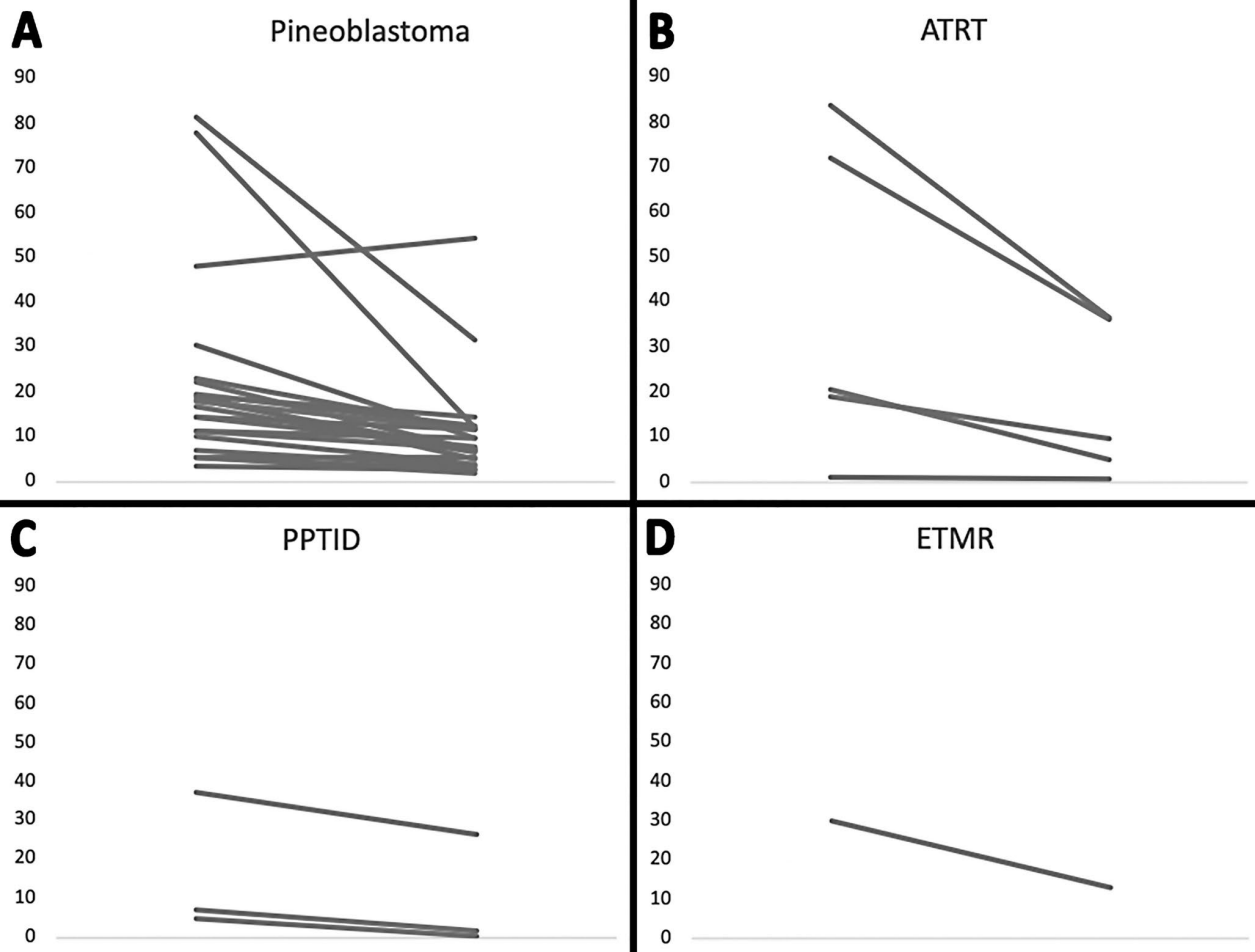
Slope plots depicting the change in tumor volume pre- and post-chemotherapy of all 31 patients. Panels **A**, **B**, **C**, and **D** show the tumor volume change in pineoblastomas, ATRTs, PPTIDs, and ETMRs, respectively. Patient 5 in Panel **A** was the only patient with tumor growth during the neoadjuvant chemotherapy course

Mean pre-chemotherapy tumor volume was 24.7 cm^3^ (range, 1.1–84.1 cm^3^), which decreased on average by 51.0% to 12.1 cm^3^ (range, 0.5–54.5 cm^3^) following chemotherapy (Table 2). This decrease resulted in an average absolute tumor volume reduction of 12.6 cm^3^ (range, -6.0–65.5 cm^3^). ATRTs (*n* = 5) were the largest tumors by volume on the initial MRI with a mean volume of 39.5 cm^3^ (range, 1.1–84.1 cm^3^), followed by ETMR (*n* = 1) at 30.1 cm^3^ (no range), pineoblastomas (*n* = 22) at 22.1 cm^3^ (range, 3.5–81.5 cm^3^), and PPTID (*n* = 3) at 16.6 cm^3^ (range, 5.0–37.3 cm^3^). ETMR, ATRTs, and pineoblastomas were the most responsive to chemotherapy with a 56.9% (no range), 54.7% (range, 10.53–74.5%), and 49.8% (range, -12.4–84.2%) volume reduction, respectively. PPTIDs had less reduction in tumor volume on average at 41.2% (range, 28.4–90.0%). Figure 1 shows the change in tumor volume for all 31 patients by tumor type.

**Fig. 2:**
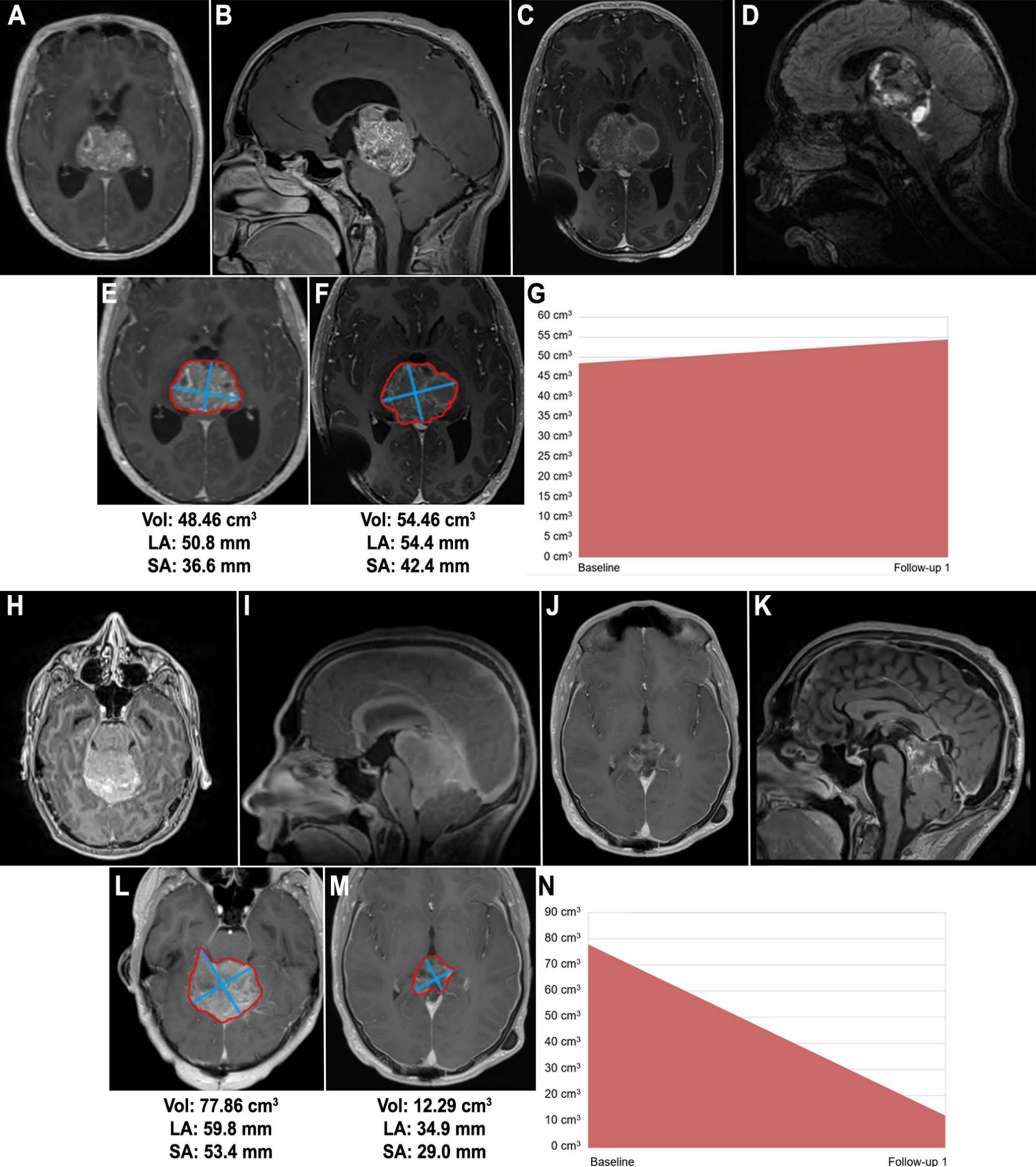
Case 1. Imaging of the only patient in our series whose tumor progressed on chemotherapy. This was a 16-year-old male with a PAG. Pre-chemotherapy (**A**) axial, and (**B**) sagittal T1-weighted with contrast MRI demonstrating the pineal mass. Post-chemotherapy (**C**) axial, and (**D**) sagittal T1-weighted with contrast MRI imaging demonstrating an increase in the volume of the mass. MRI tumor analytics in (**E**) and (**F**) show the calculated volume (cm^3^), long axis (mm), and short axis (mm) of the mass. (**G**) Visual depiction of an increase in tumor volume by 12%. Case 2. Imaging of a 22-year-old male with a pineoblastoma that decreased in size during pre-operative chemotherapy. Pre-chemotherapy (**H**) axial, and (**I**) sagittal T1-weighted with contrast MRI demonstrating a pineal mass. Post-chemotherapy (**J**) axial, and (**K**) sagittal T1-weighted with contrast MRI imaging demonstrating a decrease in the volume of the mass. MRI tumor analytics in (**L**) and (**M**) showing the calculated volume (cm^3^), long axis (mm), and short axis (mm) of the mass. (**N**) Visual depiction of a decrease in tumor volume by 65 cm^3^ (84%), the maximum absolute tumor volume reduction in our series

Only 1 patient showed tumor growth on chemotherapy—a 16-year-old boy with a large pineoblastoma/pineal anlage tumor (PAT) who exhibited a 12% increase in tumor volume after 3 cycles of chemotherapy. Maximum absolute tumor reduction after chemotherapy was 65.6 cm^3^ (pre-chemotherapy volume of 77.9 cm^3^ and post-chemotherapy volume of 12.3 cm^3^) in a 22-year-old patient with pineoblastoma (Fig. 2).

### Surgical data: operative time and extent of resection

The mean operative time was 6.2 h (range, 2.9–10.5 h). A total of 20 (64.5%) GTRs were achieved: all ATRTs (*n* = 5; 100.0%), the lone ETMR (*n* = 1; 100%), 12 of the 22 pineoblastomas (54.5%), and 2 of the 3 PPTIDs (67%). The remaining 11 cases (35.5%) were NTRs (Table 1).

### Intraoperative blood loss & transfusion requirement

The mean estimated blood loss (EBL) for all surgeries was 180 mL (range, 25–900 mL) (Table 2). On average, the mean EBL for each type of tumor was the following: pineoblastomas 182 mL (range, 25–900 mL); ATRTs 175mL (range, 100–250 mL); and PPTIDs 125 mL (range, 25–200 mL). The single ETMR had a blood loss of 275 mL (no range), ultimately requiring 200 mL of intraoperative packed red blood cells (pRBCs). Nine patients (29%) required intra-operative pRBC transfusion due to blood loss: 5 patients with pineoblastomas (22.7%); 3 patients with ATRTs (60%); and 1 patient with ETMR (100%).

The maximum recorded blood loss was 900 mL in a 16-year-old male with pineoblastoma that progressed despite 3 rounds of neoadjuvant chemotherapy—he received 3 units of pRBCs intraoperatively. The lowest estimated blood loss was 25 mL and occurred in two patients: the 6-year-old male with the PPTID that decreased by 90% following neoadjuvant chemotherapy, and a 10-year-old female with a pineoblastoma that decreased by 62% following neoadjuvant chemotherapy.

Average blood loss as a percentage of total blood volume was 11.7% (range, 1.1–38.8%). One patient with a 39% reduction in estimated total blood volume was a 14-month-old, 8.6 kg female with a pineoblastoma. She received 2 rounds of neoadjuvant chemotherapy and experienced an EBL of 250 mL, for which she was given 170 mL of pRBCs. Her tumor decreased from 17.9 cm^3^ to 11.8 cm^3^ during chemotherapy.

### Intraoperative tumor findings

Upon review of the operative reports, 18 (58.1%) of the 31 tumors were explicitly described as “avascular” or “hypovascular,” 6 (19.4%) were noted to be largely necrotic, 9 (29.0%) were described as “soft and easily aspirated,” and 3 (9.7%) were described as “mixed density.” Aiding in resection and manipulation of the tumor, 8 (25.8%) tumors were described as having a well-defined capsule, and 7 (22.6%) operative reports commented on a “fibrotic” consistency of capsule and tumor. The fibrotic walls of the tumor were sometimes adherent to the deep venous structures, but resection was easier because of less bleeding from the tumor and sharp dissection of the capsule.

### Clinical data: length of stay and readmissions

The mean length of stay in the intensive care unit (ICU) was 2.8 days (range, 1.0–10.0 days) and the mean hospital length of stay (LOS) was 6.3 days (range, 2.0–14.0 days). Five patients were readmitted to the hospital following tumor resection within 90 days: 4 within 30 days and 1 between 31 and 90 days. One patient was readmitted for seizures a week after discharge, which were effectively controlled with a single agent. Two patients were readmitted 2 weeks following discharge for pseudomeningocele repair before resuming chemotherapy. Both were discharged without further complications. One patient was readmitted 3 weeks following discharge for shunt malfunction and revision; he was discharged the following day without complications. One patient returned to the hospital 77 days following discharge for somnolence with suspected shunt malfunction. The shunt was found to be functioning, so the patient was observed overnight and improved. He was discharged the following day.

### Post-operative events and neurologic deficits

During the index hospitalization, 2 patients required a return to the operating room—one returned for pseudomeningocele repair on postoperative day 5 and another for a shunt placement on postoperative day 9.

Ophthalmoparesis, an expected new neurologic finding, was observed in 10 patients, including 7 with Parinaud’s syndrome (i.e., isolated upward gaze palsy with other clinical features such as nystagmus), 1 of whom also demonstrated a homonymous hemianopsia due to retraction of the occipital lobe via an occipital interhemispheric transtentorial approach. At last follow-up, this patient’s homonymous hemianopsia had improved but did not fully recover. Parietal lobe retraction-related contralateral motor deficits, also an anticipated postoperative deficit, developed in 5 patients who all underwent parietal interhemispheric transcallosal intervenous approaches [19]. One patient had hemiparesis and 4 had isolated lower extremity weakness. All patients had near or full recovery of their weakness at last follow-up. Two patients developed postoperative seizures, both of whom were successfully managed with antiepileptic medication.

### Survival

At the last follow-up, 20 patients (64.5%) were alive with an average length of follow-up of 6.3 years (range, 1.0–14.0 years). Of these patients, 2 had tumor recurrence or progression of residual disease; the remaining 18 patients had either stable residual tumor versus postoperative change or remained disease free. Eleven patients (35.4%) died—7 patients with pineoblastomas and 4 with ATRTs. The mean overall survival of these 11 patients was 1.9 years (range, 0.3–4.8 years); the mean was 2.1 years (range, 0.7–4.8 years) for those with pineoblastoma, and 1.5 years (0.3–3.1 years) for those with ATRT.

## Discussion

**Fig. 3:**
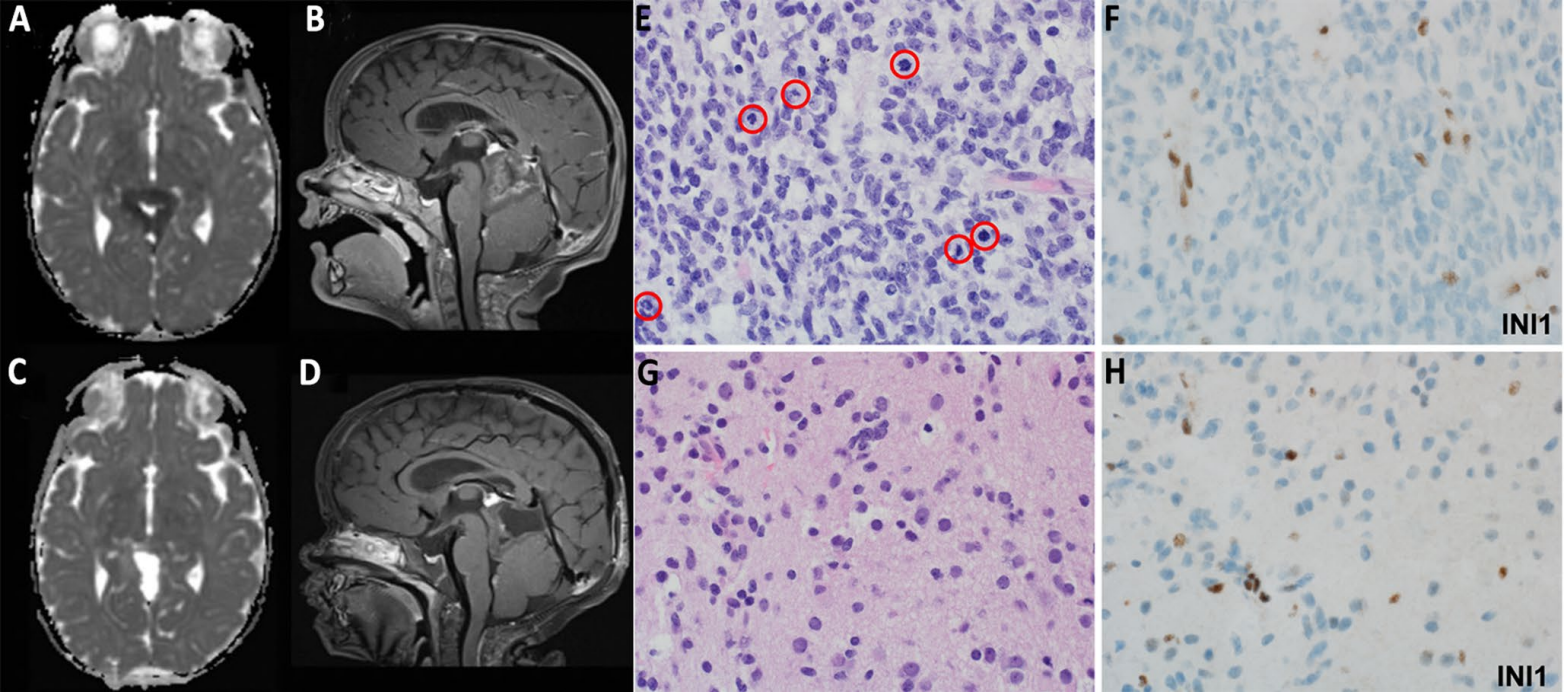
Imaging of an 11-year-old female with an ATRT that underwent biopsy and 2 rounds of neoadjuvant therapy prior to resection. (**A**) Axial and (**B**) sagittal pre-chemotherapy MRIs demonstrate a large pineal region mass compressing the superior aspect of the fourth ventricle. (**C**) Axial and (**D**) sagittal post-chemotherapy MRIs demonstrate the same pineal region mass that is smaller, less solid, more cystic, less intensely enhancing, and contains less restricted diffusion than the pre-chemotherapy imaging. Histopathologic features of this child’s tumor before and after chemotherapy. (**E**) Diagnostic biopsy reveals a hypercellular embryonal tumor with frequent mitotic figures (red circles). (**F**) Immunohistochemistry shows loss of nuclear INI1 (SMARCB1) expression in the neoplastic cells, with retained internal positive control in non-neoplastic cells. (**G**) Post-neoadjuvant chemotherapy resection shows a significant reduction in tumor cellularity and mitotic activity. (**H**) Loss of INI1 expression remains consistent in the post-treatment sample, confirming the diagnosis

Our institutional experience suggests that neoadjuvant chemotherapy represents an effective and safe adjunct in the management of embryonal pediatric pineal region tumors. All but one of the patients in this series experienced tumor volume reduction; on average, tumor size decreased by half, and a subset of patients experienced particularly dramatic results. We also found that neoadjuvant therapy resulted in notable tumoral changes, including relative hypovascularity and hypocellularity, cystic degeneration, necrosis, and a more fibrotic or firm consistency with a well-developed capsule (Fig. 3). It stands to reason that smaller and less vascular tumors should be technically easier to remove. Indeed, Schulz et al. noted that endoscopic and microsurgical resections of smaller, less vascular lesions in the pineal region yielded improved functional outcomes and fewer complications [7]. Therefore, neoadjuvant chemotherapy *may* result in a safer and less technically demanding tumor resection.

Although there are many published surgical series on pineal region tumors, there is surprisingly little information regarding the use of neoadjuvant therapy. In one of the largest cohorts, Cavalheiro et al. presented their experience in 151 children over 29 years but made no mention of the use of neoadjuvant chemotherapy [14]. Other studies also make no mention of neoadjuvant chemotherapy, including Szathmari et al. (151 patients over a 23-year study period), Tomita et al. (92 patients across 29 years), Pettorini et al. (24 patients across 12 years), Malik et al. (56 patients across 25 years), and even the consensus Society of Neuro-Oncology (SNO) review article by Liu et al. [3, 6, 12, 13, 15]. Therefore, the literature would suggest that neoadjuvant chemotherapy for pineal region tumors is a strategy that is either unknown to most or rarely utilized. Indeed, we found only a few patients reported in the literature. In their systematic review of preoperative chemotherapy in pediatric brain tumors, Arguello et al. included 6 patients from their own institution (Texas Children’s Hospital, Houston), 1 of whom was a 2-year-old with a large pineoblastoma [16]. The child’s tumor shrank dramatically after 2 cycles of vincristine and cyclophosphamide followed by a subsequent GTR; unfortunately, the patient died 19 months later. The authors felt that contrast-enhancing tumors may be more likely to respond to chemotherapy than non-enhancing tumors [16]. In an article on second-look surgery for pineal region tumors, Ogiwara reported 2 children (both 1.4 years of age) with ATRTs that experienced reduction in tumor size with chemotherapy followed by GTRs [20]. Similarly, Iwama et al. reported a 2-year-old male with an ATRT and a 3-year-old female with an ETANR who had a similar course as those in Ogiwara’s study [21].

All patients in our series had either a GTR or NTR. Intraoperative blood loss was still substantial in certain cases, and intraoperative blood products were administered in about a third of the patients (29%). Nonetheless, the average blood loss was 11.7% of total blood volume, and no surgery had to be prematurely terminated because of excessive intraoperative blood loss. Furthermore, there were no complications due to the pancytopenic effects from chemotherapy and no drug-induced toxicities.

Others have observed similar effects of cytoreductive chemotherapy in other types of brain tumors, such as the cystic and fibrotic transformation of the tumor, which can improve the surgical peritumoral planes of dissection and the intratumoral vascular environment [4, 16, 17]. In the pineal region, however, we observed that post-chemotherapy, the tumor vascularity is favorable, but the tumor consistency is often more fibrous. This contrasts with chemotherapy-naive embryonal tumors that are typically soft and easily aspirated with a hand-held sucker but can be bloody. On the one hand, the fibrotic nature of the tumor and surrounding neocapsule that results from neoadjuvant chemotherapy allows the surgeon to pull or peel tumor off surrounding structures (e.g., tectum) and into the operative field. On the other hand, the fibrotic transformation may cause the tumor to be more adherent to the already naturally thick arachnoid of the pineal region and the network of veins that often surround these tumors. Studies by Tomita et al. and Pettorini et al. have underscored that the proximity of these veins often dictates the extent of safe resection [6, 15]. In our opinion, sharp dissection is necessary in these more adherent areas, with careful attention to minimizing any traction on these thin-walled veins.

Analogous benefits have been established in other high-grade, hypervascular pediatric brain tumors, such as choroid plexus carcinoma, ATRT, and ETMR. For CPCs, several studies have demonstrated that neoadjuvant chemotherapy can convert a subtotal resection into a GTR by shrinking the tumor and reducing vascularity, resulting in improved survival outcomes [4, 18, 19]. Likewise, neoadjuvant regimens in ATRT and ETMR have been associated with improved resection safety, decreased perioperative morbidity, and enhanced local control [12, 13]. The parallels between these tumor types and pineal-origin embryonal lesions provide a strong biological rationale for a similar multimodal approach.

### Study strengths and limitations

This is a unique series of patients from a single institution who harbored pineal region tumors and underwent at least 2 rounds of chemotherapy prior to definitive resection. It adds to the limited literature on this topic. We provide detailed objective volumetric analysis of the effect that chemotherapy had on the tumor. While it remains to be seen whether our results and experiences are replicated by others, the chemotherapy given to these patients is a straightforward “one size fits all” regimen, regardless of tumor pathology. We recognize that intraoperative blood loss and even need for transfusion are, to some degree, subjective outcomes. Finally, a concurrent, control group (matched on age, tumor pathology and volume) would have been ideal, but given the rarity of these tumors and our bias to use neoadjuvant chemotherapy, such a control group was not possible. We are currently contemplating a multi-institution study to expand on our work.

## Conclusions

In our series, neoadjuvant chemotherapy for pineal region tumors (predominantly embryonal) safely reduced tumor volume in almost all patients. This reduction in tumor burden and blood supply may result in better surgical outcomes. Further studies, ideally across multiple institutions, are needed to validate our findings.

## Data Availability

No datasets were generated or analysed during the current study.
